# *Schizosaccharomyces pombe* Ofd2 Is a Nuclear 2-Oxoglutarate and Iron Dependent Dioxygenase Interacting with Histones

**DOI:** 10.1371/journal.pone.0025188

**Published:** 2011-09-16

**Authors:** Hanne Korvald, Anne Margrethe Mølstad Moe, F. Henning Cederkvist, Bernd Thiede, Jon K. Laerdahl, Magnar Bjørås, Ingrun Alseth

**Affiliations:** 1 Department of Microbiology, Oslo University Hospital HF Rikshospitalet, Oslo, Norway; 2 Centre for Molecular Biology and Neuroscience (CMBN), University of Oslo, Oslo, Norway; 3 Department of Medical Biochemistry, University of Oslo, Oslo, Norway; 4 The Biotechnology Centre of Oslo, University of Oslo, Oslo, Norway; Tulane University Health Sciences Center, United States of America

## Abstract

2-oxoglutarate (2OG) dependent dioxygenases are ubiquitous iron containing enzymes that couple substrate oxidation to the conversion of 2OG to succinate and carbon dioxide. They participate in a wide range of biological processes including collagen biosynthesis, fatty acid metabolism, hypoxic sensing and demethylation of nucleic acids and histones. Although substantial progress has been made in elucidating their function, the role of many 2OG dioxygenases remains enigmatic. Here we have studied the 2OG and iron (Fe(II)) dependent dioxygenase Ofd2 in *Schizosaccharomyces pombe*, a member of the AlkB subfamily of dioxygenases. We show that decarboxylation of 2OG by recombinant Ofd2 is dependent on Fe(II) and a histidine residue predicted to be involved in Fe(II) coordination. The decarboxylase activity of Ofd2 is stimulated by histones, and H2A has the strongest effect. Ofd2 interacts with all four core histones, however, only very weakly with H4. Our results define a new subclass of AlkB proteins interacting with histones, which also might comprise some of the human AlkB homologs with unknown function.

## Introduction

2-oxoglutarate (2OG) and iron (Fe(II)) dependent dioxygenases constitute a huge superfamily of enzymes found throughout biology. The reactions driven by these dioxygenases are carried out in the presence of oxygen, 2OG as cosubstrate and Fe(II) as cofactor. Oxygen is consumed in the reaction and 2OG is decarboxylated to yield succinate and carbon dioxide [1]–[3]. In some cases, like for collagen prolyl hydroxylase, ascorbate is required for optimal activity [4]. Although this superfamily of enzymes is one of the most diverse, the members hold a highly conserved Fe(II) binding HXD/E…H triad motif [1], [5]. The 2OG binding residues are less conserved and are characteristic of each subfamily. Substrate binding residues vary as well and may involve structurally flexible segments surrounding the active site [1].

2OG/Fe(II) dioxygenases catalyze a wide range of biological reactions involving a multitude of substrates [3], [6]. Among these reactions are collagen biosynthesis, fatty acid metabolism, hypoxic sensing, and histone and nucleic acid demethylations. Prolyl hydroxylases, involved in cellular responses to hypoxia, hydroxylate proline residues of the hypoxia-inducible transcription factor (HIF) [7], [8]. Several Jumonji C (JmjC) domain-containing enzymes hydroxylate methylated lysine and arginine residues of histones resulting in demethylation [9], [10], whereas AlkB enzymes are engaged in demethylation of nucleic acid substrates [11]. Given the diversity of this group of enzymes, elucidating the function of the yet uncharacterized 2OG/Fe(II) dioxygenases is a challenging task.

The AlkB dioxygenase from *Escherichia coli* is a repair enzyme which removes alkyl groups from DNA and RNA by direct reversion. Preferred substrates are 1-methyladenine (1 mA) and 3-methylcytosine (3 mC) in single-stranded DNA and RNA [12], [13]. In addition, AlkB repairs 1-methylguanine and 3-methylthymine [14], [15], as well as exocyclic etheno (i.e. εA and εC) and ethano adducts [16]–[19]. Nine human homologs (ALKBH1-8), including the fat mass and obesity associated protein FTO, are known. *In vitro* DNA repair activities have been published for ALKBH1 [20], ALKBH2, ALKBH3 [21] and FTO [22], but only ALKBH2 has been thoroughly investigated *in vivo* and is broadly accepted as a true AlkB repair enzyme [23], [24]. ALKBH8 consists of both a methyltransferase domain and an AlkB domain, and these respective activities are recently described on wobble nucleosides in mammalian tRNA [25]–[28].

A set of 2OG/Fe(II) dioxygenases are known to be hypoxia-inducible and among these are HIF hydroxylases and several JmjC histone demethylases. Recently, Hughes and Espenshade published that expression of the fission yeast *Schizosaccharomyces pombe* 2OG/Fe(II) dioxygenase 1 (*ofd1* ^+^) gene is upregulated under low oxygen and that Ofd1 is involved in degradation of sterol regulatory element binding protein Sre1 [29]. Microarray analysis revealed a second 2OG/Fe(II) dioxygenase as being Sre1 dependent and hypoxia-inducible [5], [30] and this second gene, *ofd2* ^+^, is described in the present work. We show that Ofd2 catalyzes the formation of succinate in a typical hydroxylation reaction that is dependent on 2OG and Fe(II). Furthermore, succinate formation by Ofd2 is stimulated by the presence of histones, and we find specific interactions between Ofd2 and histones. Ofd2 is categorized as an AlkB homolog; however, Ofd2 does not exhibit any AlkB-like DNA repair activity nor is the *ofd2* ^−^ mutant sensitized when exposed to DNA damaging agents.

## Results

### Identification and bioinformatics analysis of *S. pombe* Ofd2

It was recently shown that expression of the 2OG/Fe(II) dioxygenases *ofd1* ^+^ and *ofd2* ^+^ is upregulated under low oxygen and dependent on the transcription factor Sre1 [29]. Whereas Ofd1 is involved in degradation of Sre1, the function of Ofd2 is unknown.

**Figure 1:**
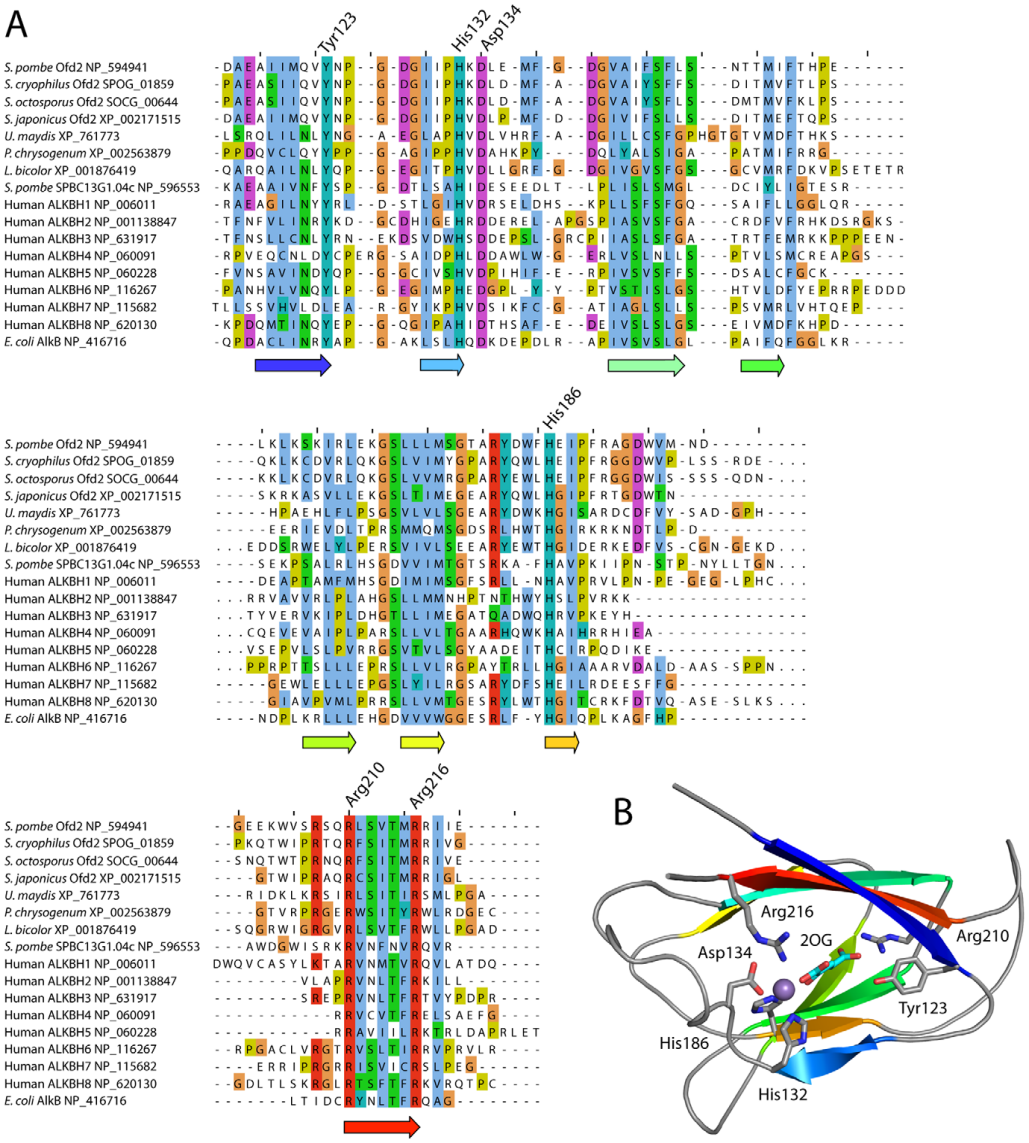
*Schizosaccharomyces* Ofd2 belongs to the AlkB subfamily of 2OG/Fe(II) dioxygenases. (**A**) A multiple sequence alignment (MSA) of the 2OG/Fe(II) dioxygenase core segment of *S. pombe* Ofd2 (residues 114–220) and homologous sequences from three other *Schizosaccharomyces* species, the fungi *Ustilago maydis*, *Penicillium chrysogenum*, and *Laccaria bicolor*, *S. pombe* SPBC13G1.04c, human ALKBH1-8, and *E. coli* AlkB, show that Ofd2 is an AlkB family member with conserved metal and 2OG binding residues. The eight β-strands of the *E. coli* AlkB core domain, shown as arrows below the MSA, are conserved in Ofd2. Gaps and deleted sequence are shown as dashes and dots, respectively. NCBI sequence identifiers [43] are shown where these are available. (**B**) A structural model of the *S. pombe* Ofd2 2OG/Fe(II) dioxygenase core shows the ‘jellyroll’ structural fold with the eight β-strands (same coloring as in panel A) and the location of the conserved 2OG (cyan sticks) and Fe(II) (purple ball) complexing residues.

Bioinformatics analysis reveals that the 678 nucleotide *ofd2* ^+^ gene (SPAP8A3.02c) has no introns and translates into a protein of 225 amino acids. The calculated molecular mass is 25.9 kDa and the isoelectric point (pI) is 4.5. Amino acid sequence profile analysis and a multiple sequence alignment of Ofd2 and other AlkB homologs show that the 2OG/Fe(II) core domain corresponds to residues 116–216 of Ofd2 (Figure 1A). This domain has a ‘jellyroll’ structural fold built from eight β-strands (Figure 1B) [31]. Structural disorder predictions suggest that the 30–35 N-terminal residues of Ofd2 are structurally disordered, while residues 36–115 are structurally ordered, but at present with unknown structure. The consensus sequence for Fe(II) coordination by 2OG/Fe(II) dioxygenases is HXD/E…H [1], [5], which is conserved in Ofd2 as His132, Asp134 and His186 (Figure 1A), and we predict that Ofd2 coordinates Fe(II) with these residues (Figure 1B). Also, conserved in Ofd2 is Tyr123 of the first AlkB core β-strand, and the two arginine residues of the C-terminal β-strand, Arg210 and Arg216, which form stabilizing salt bridges with the carboxylate groups of 2OG (Figure 1).

A reliable phylogeny for the eukaryotic AlkB clade of the 2OG/Fe(II) dioxygenases has not yet been described and is far from trivial to generate due to the very limited sequence conservation and large number of paralogs in many species. In addition to Ofd2, the *S. pombe* genome encodes a second AlkB homolog (SPBC13G1.04c) which is not yet experimentally characterized. This open reading frame is clearly more similar to human ALKBH1 than to the other human AlkB homologs and has significantly better similarity to *E. coli* AlkB than Ofd2. Ofd2, on the other hand, does not show strong similarity to any of the human AlkB homologs, and might represent a unique fungal evolutionary clade of AlkB homologs.

Comparison of *S. pombe* Ofd2 with *E. coli* AlkB, for which the 3D structure has been solved [31], [32], revealed that the catalytic core characteristic of 2OG/Fe(II) dioxygenases is conserved in Ofd2 (Figure 1B). The AlkB structure has a unique nucleotide-recognition lid within the N-terminal sequence segment [31]. The lid is a β-sheet-forming subdomain that contacts the nucleotide substrate and covers the active site of AlkB. A reliable multiple sequence alignment of the N-terminal segments of AlkB, Ofd2 and other AlkB homologs was not possible to generate due to low sequence similarity. However, the structurally ordered part of the N-terminus of Ofd2 is approximately 35 residues shorter than the N-terminus of AlkB, suggesting that Ofd2 has a smaller lid and a more open active site that could accommodate a more bulky substrate than AlkB.

### Ofd2 is a nuclear 2OG/Fe(II) dioxygenase

**Figure 2:**
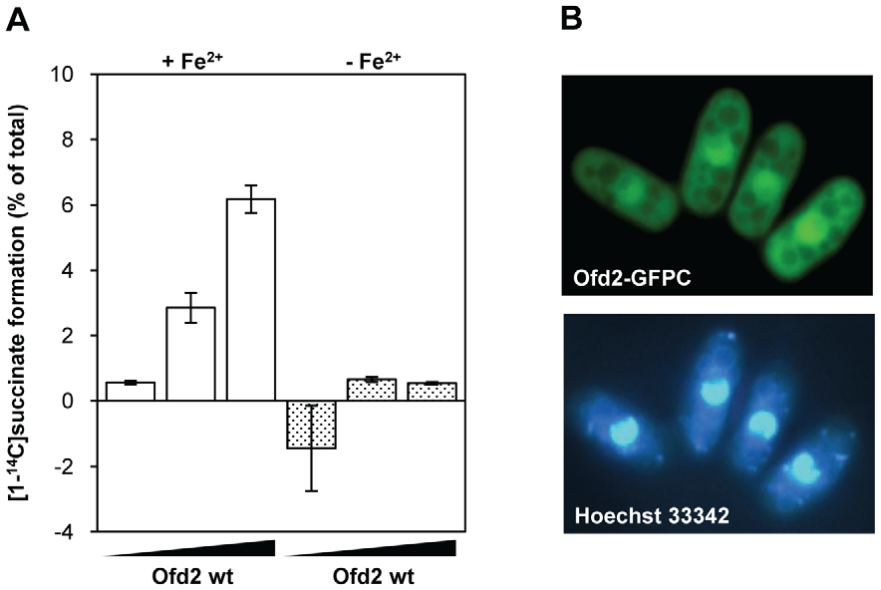
Ofd2 is a nuclear 2OG/Fe(II) dioxygenase. (**A**) Ofd2 (from left to right: 35, 200, 350 pmol) was incubated with 2-oxo[5-^14^C]glutarate and the resulting [1-^14^C]succinate formation (white bars: +Fe(II); dotted bars: −Fe(II)) was measured by scintillation counting. Results are means ± standard deviation (SD) of duplicate samples. (**B**) Fluorescence microscopy of *S. pombe* cells expressing GFP-tagged Ofd2 (upper panel) and control staining of nucleus with Hoechst 33342 (lower panel).

A feature of many 2OG/Fe(II) dioxygenases is that they can oxidize 2OG to carbon dioxide and succinate in the absence of their prime substrate [12], [13], [33]. To test whether *ofd2* ^+^ encodes an active dioxygenase, the full length protein was expressed in *E. coli* and purified to apparent homogeneity as determined from SDS-polyacrylamide gel analysis. An assay was employed that measures the production of [1-^14^C]succinate from [5-^14^C]2OG. This assay demonstrated that Ofd2 stimulated succinate release in a manner that was dependent on supplementation with Fe(II), thus indicating that Ofd2 is an active 2OG dioxygenase (Figure 2A).

To search for potential substrates for Ofd2, intracellular sorting was studied. *ofd2* ^+^ was cloned in front of the gene encoding green fluorescent protein, *GFP* ^+^, to yield Ofd2-GFP fusion protein for ectopic expression in *S. pombe* cells. Ofd2-GFP accumulated in the nucleus and was excluded from the nucleolus (Figure 2B). Some cytoplasmic staining was evident in a number of cells, which probably reflects overexpression of Ofd2-GFP. This result is in agreement with results from the ORFeome Localization Project [34], which also found nuclear sorting of Ofd2 in addition to some cytoplasmic staining (*S. pombe* Postgenome Database service, ORFeome Localization data, Chemical Genetic Service, Riken, Japan). To verify that the GFP signal was derived from Ofd2-GFP fusion, western blot analysis of whole cell protein extracts made from Ofd2-GFP overexpressing cells, was performed. Immunoblot probed with Ofd2 antibody revealed that Ofd2 existed as a fusion protein (Figure S1). Also with a GFP antibody the major signal was full-length Ofd2-GFP and a minor fraction corresponded to free GFP (Figure S1).

Nuclear localization of Ofd2 and its homology to *E. coli* AlkB could imply a role for Ofd2 in removal of DNA damage. Ofd2 was tested for activity against different DNA lesions and AlkB was included as a positive control. Release of [^3^H]-methylated bases from single and double stranded DNA oligonucleotides containing 1 mA and 3 mC was measured (Methods S1) and AlkB was active as expected. However, no activity was detected for Ofd2, neither towards the alkylated bases (Figure S2A and S2B) nor a substrate containing εA (data not shown). This result was supported by analysis of an *S. pombe ofd2^−^* mutant (Methods S1). When challenged with the alkylating agent methyl methanesulfonate (MMS), which sensitizes the *E. coli alkB* mutant, the *ofd2^−^* mutant behaved as the wild type cells (Figure S3).

### Specific interactions between Ofd2 and histones

**Figure 3:**
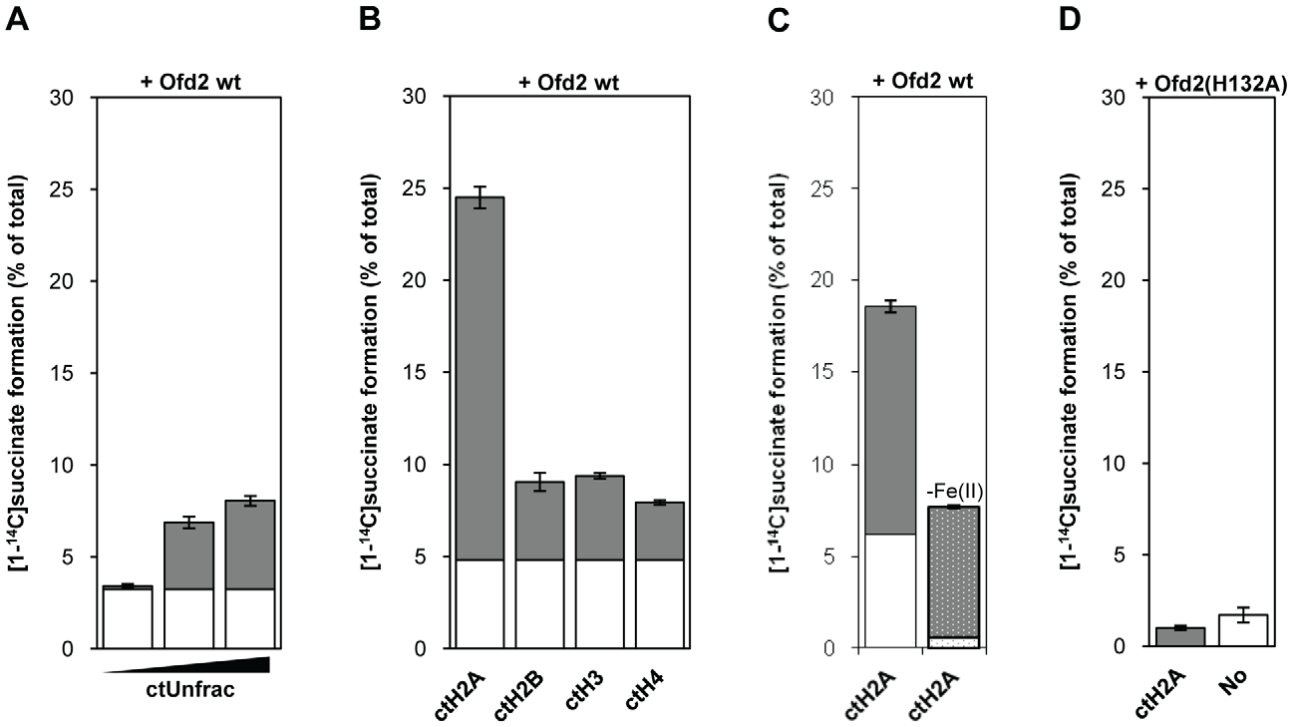
The decarboxylase activity of Ofd2 is stimulated by histones. Ofd2 was incubated with [5-^14^C]2OG in the presence or absence of histones and the resulting [1-^14^C]succinate formation was measured by scintillation counting. Results are means ± SD of duplicate samples. The average basal level (Ofd2 without histones) is shown as white bars in front of the grey bars, which show succinate release stimulated by histones. (**A**) 200 pmol Ofd2 in the presence or absence of unfractionated calf thymus histones (from left to right: 1, 4, 16 µg). (**B**) 350 pmol Ofd2 in the presence or absence of 700 pmol calf thymus histones (from left to right: ctH2A, ctH2B, ctH3, ctH4). (**C**) 350 pmol Ofd2 in the presence or absence of 350 pmol ctH2A. The dotted bar is without Fe(II). (**D**) 350 pmol Ofd2(H132A) in the presence or absence of 700 pmol ctH2A.

In addition to acting on nucleic acid substrates, 2OG/Fe(II) dioxygenases are engaged in protein modifications such as hydroxylation of prolyl residues of collagen, prolyl and asparaginyl residues of HIF, asparaginyl and aspartyl residues in epidermal growth factor (EGF), as well as methylated lysyl and arginyl residues of histones. Given the low pI for Ofd2, which generates a negative protein outer surface, positively charged histones could be appropriate substrate candidates. To test this, succinate release was measured after incubation of Ofd2 with unfractionated calf thymus (ct) histones. Ofd2 activity was stimulated by addition of histones, and succinate formation increased linearly with increasing concentrations of histones (Figure 3A). Next, Ofd2 was assayed with each of the four core histones and ctH2A stimulated succinate release by Ofd2 4–5 fold stronger as compared to ctH2B, ctH3 and ctH4 (Figure 3B). Withdrawal of iron from the reaction mixture with ctH2A reduced the Ofd2 activity 60% (Figure 3C). The rest activity could be due to the presence of Fe(II) in the recombinant Ofd2 protein. Further, substitution of the first histidine in the iron binding motif (His132) to an alanine residue totally abolished the succinate formation activity (Figure 3D), confirming that the activity measured was inherent to Ofd2 and dependent on an intact HXD/E…H triad motif.

To further characterize Ofd2, activity assays were performed with recombinant (r) human H2A and also H2A peptides corresponding to residues 1–100 or 100-end. Under these conditions, no stimulation of 2OG turnover by Ofd2 was observed (data not shown). Neither did recombinant *S. pombe* H2A.1 and H2A.2 stimulate Ofd2 activity, excluding species variations as the reason for the negative result. In summary, these results suggest that one or more of the modifications present on the ct-histones are essential for Ofd2 activity.

The JmjC family members of 2OG/Fe(II) dioxygenases are all engaged in demethylation of lysine or arginine residues on histones; however, to date, methylations have been demonstrated for H2B, H3 and H4, but not H2A. To search for methylations, ctH2A was subjected to LTQ-Orbitrap mass spectrometry (MS) analysis after treatment with one of the proteases trypsin, Lys-C or chymotrypsin. Sequence coverage varied from 15 to 60%. The MS analysis did not reveal any methylated residues, suggesting that Ofd2 is not involved in demethylation of ctH2A. As 2OG/Fe(II) dioxygenases perform hydroxylation reactions and Ofd2 has been linked to prolyl hydroxylases [29], ctH2A was also analyzed for proline hydroxylations. However, no differences with or without Ofd2 treatment were found.

**Figure 4:**
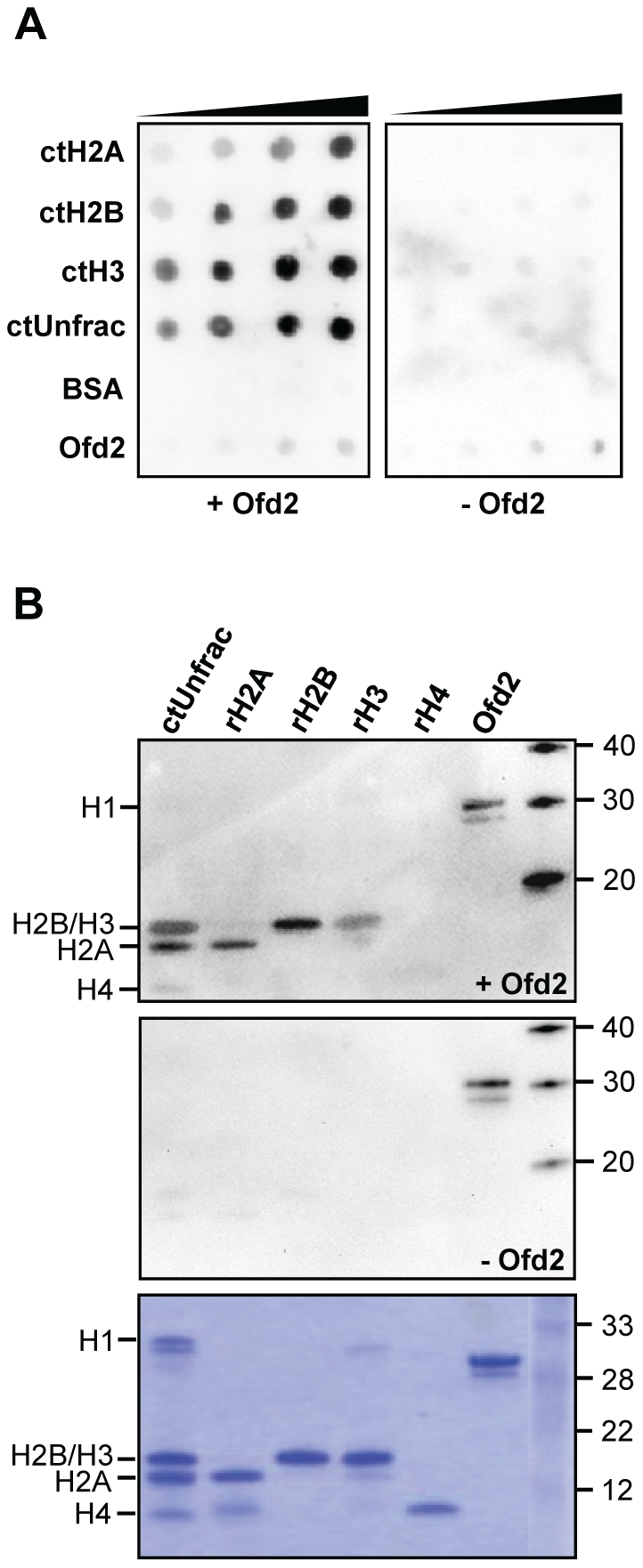
Ofd2 interacts with histones. (**A**) Dot blot analysis. Calf thymus histones (ctH2A, ctH2B, ctH3 and unfractionated whole histones), BSA and Ofd2 were dotted onto a PVDF membrane and probed with Ofd2 (left panel) or with no protein (right panel). Protein complexes were visualized with chemiluminescence. (**B**) Far western analysis. Unfractionated calf thymus histones, recombinant histones (rH2A, rH2B, rH3, rH4) and Ofd2 were separated on a 12% SDS-PAGE, transferred to a PVDF membrane and probed with Ofd2 (upper panel) or with no protein (middle panel). A third gel was stained with Coomassie Blue to visualize the proteins (lower panel).

Motivated by the stimulation of 2OG turnover by histones, the potential binding between Ofd2 and histones was analyzed. Dot blot revealed interactions between Ofd2 and ctH2A, ctH2B, ctH3 and unfractionated ct-histones, but not with bovine serum albumin (BSA) as control (Figure 4A, left panel). A similar blot not probed with Ofd2 (Figure 4A, right panel) showed only a weak background signal in addition to the Ofd2 control. Since ctH4 could no longer be purchased, the unfractionated ct-histones were separated by SDS-PAGE and far western analysis performed. H4 is the smallest of the histones and migrates in front on the gel. Ofd2 did also bind denatured ct-histones, and the affinity for ctH4 was less than for the other three histones. Recombinant histones were included in the far western analysis, and Ofd2 interacted with rH2A, rH2B and rH3, whereas only a very weak signal was obtained for rH4 (Figure 4B). In conclusion, although Ofd2 2OG turnover is stimulated most strongly by ctH2A, Ofd2 seems to bind all histones (calf thymus and recombinant), however, with less affinity for H4.

If histone methyl groups are the target for Ofd2, one might expect that yeast cells lacking Ofd2 would have a different euchromatin/heterochromatin status than corresponding wild type cells, as seen for the *epe1* ^−^ fission yeast mutant [35]. To check for silencing effects, the *ofd2* ^−^ mutant was crossed into four reporter strains containing the *ura4^+^* gene in different heterochromatic loci: repeats of centromere 1 [*imr1*R(*Nco*I)::*ura4* ^+^], central core of centromere 2 [*cen2*(*Sph*1)::*ura4* ^+^], mating-type region [*mat3-M*(*Eco*RV)::*ura4* ^+^] and ribosomal DNA [rDNA::*ura4* ^+^]. Expression of the *ura4^+^* gene was estimated by plating serial dilutions of cells on selective plates (Methods S1). The *ofd2^−^* strains grew poorly on plates lacking uracil but formed colonies on plates containing fluoroorotic acid (FOA) similar as the wild type strains (data not shown), suggesting that Ofd2 is not necessary for transcriptional silencing.

## Discussion

2OG/Fe(II) dioxygenases are involved in a wide range of biological processes and catalyze oxidation reactions in the presence of Fe(II) by the use of oxygen and 2OG. Here, we show that Ofd2 from fission yeast mediates decarboxylation of 2OG in the absence of a primary substrate. This activity is dependent on Fe(II) and an intact HXD/E…H motif, demonstrating that Ofd2 is a true 2OG and Fe(II) dependent dioxygenase.

Sequence comparisons revealed that Ofd2 belongs to the AlkB family of dioxygenases. *E. coli* AlkB, and also human ALKBH2 and ALKBH3, remove methyl damages from DNA and RNA bases, thereby restoring correct base pairing properties. We were not able to detect DNA repair activities for Ofd2, as previously observed [36]. This result was supported by survival analysis of the *ofd2^−^* mutant, suggesting that Ofd2 is not a functional AlkB homolog. Further, ALKBH8 was recently shown to modify wobble nucleosides in certain tRNAs [26], [27]. However, nuclear localization of Ofd2 is in disagreement with a function of Ofd2 similar to ALKBH8, which is localized to the cytoplasm [37] where tRNA modifications are carried out. Further, analysis of *S. pombe* tRNA have shown that the wobble nucleoside (*S*)-5-methoxycarbonylhydroxymethyluridine ((*S*)-mchm^5^U) which is the product of ALKBH8 hydroxylation, is absent in fission yeast [25]. However, the precursor 5-methoxycarbonylmethyluridine (mcm^5^U) was identified and a tRNA methyltransferase (Trm9; SPAC13D6.03c), with homology to the methyltransferase domain of ALKBH8, is present in the *S. pombe* genome, demonstrating that at least parts of the tRNA modification apparatus found in higher eukaryotes is intact in fission yeast.

Succinate formation by Ofd2 was stimulated when incubated with histones, especially H2A, suggesting that histones could be the prime substrate. However, despite thorough examination by MS, no change in modification pattern was detected. We speculate that another unknown biomolecule is the target for the oxidation reaction. If Ofd2 acts in a trans mode, one of the other histones in the nucleosome core could be the target molecule. Alternatively, additional proteins or cofactors might be necessary for the complete reaction to take place.

Another class of 2OG/Fe(II) dioxygenases, the JmjC family, was shown to possess histone demethylation activity [38] by the use of the same mechanism as AlkB demethylation of DNA. In *S. pombe*, seven JmjC proteins have been identified and in humans about 30 which can be grouped into seven distinct subfamilies. The JmjC proteins have a very different sequence signature compared to AlkB and Ofd2 does not belong to this family. Hence, this is the first demonstration of an AlkB homolog that is stimulated by addition of protein and, moreover, that interacts with histones. Ofd2 was neither stimulated by recombinant histones nor *in vitro* synthesized peptides, probably suggesting that one or more specific histone modifications are required for the correct interaction to take place.

If histones are the favored substrate for Ofd2, we speculate that Ofd2 is involved in up- or downregulation of transcription. However, assays with the *ura4* ^+^ reporter strains did not reveal this. In all four reporter strains, the *ura4* ^+^ gene was placed in heterochromatic regions, and it might be that Ofd2 rather is involved in modulation of euchromatin in transcription, replication, repair or other DNA transactions. The *ofd2* ^+^ gene itself is upregulated in response to hypoxia, which could imply a role in regulation of cellular responses to hypoxia. Among the human AlkB homologs, only ALKBH5 is upregulated in response to hypoxia [39]. ALKBH5 was also nuclear and shown to possess uncoupled decarboxylation of 2OG but the prime substrate was not identified. It remains to be investigated whether ALKBH5, or any of the other human AlkB homologs with unknown substrate, interact with and/or have histones as their primary substrate.

## Materials and Methods

### Reagents

Calf thymus histones were from Sigma (Unfractionated whole histone: H9250) and Roche (H2A: 11034740001, H2B: 10223514001, H3: 11034758001, H4: 10223492001). The quality and quantity of the histone preparations were confirmed by SDS-PAGE. None of these, except H3, are now commercially available. Human recombinant histones were from New England Biolabs (H2A: M2502, H2B: M2505, H3.1: M2503, H4: M2504). Human H2A peptides 1–100 (ab27894) and 100-end (ab15660) were from Abcam. Recombinant *S. pombe* H2A.1 and H2A.2 were purified from *E. coli* by a 3-step procedure including Ni-NTA agarose, Superdex 75 and Resource S chromatography.

### Expression and purification of recombinant wild type and mutant Ofd2

For expression and purification of Ofd2 in *E. coli* the gene sequence of *ofd2* ^+^ was PCR amplified using the primers 5′-CATGCCATGGTC**CATATG**TTGTATGAAAATATGAGC-3′ and 5′-CG**GGATCC**TTATCCAAAAACATGATTTTC-3′ (restriction sites in bold letters). The PCR product was exchanged with the *Nde*I-*Bam*HI fragment of a pET28b vector (Novagen), in-frame with an N-terminal 6xHIS-tag. The Ofd2(H132A) mutant was constructed by site-directed mutagenesis using QuikChange site-directed mutagenesis kit (Stratagene/Agilent Technologies) with the primers 5′-CCAGGTGATGGAATTATACCA**GC**TAAAGACTTAGAAATGTTTGG-3′ and 5′-CCAAACATTTCTAAGTCTTTA**GC**TGGTATAATTCCATCACCTGG-3′ and pET28b-Ofd2 as a template. The CA→GC mutation in the primers is shown in bold letters. Correct mutant construct was confirmed by sequencing. *E. coli* BL21-CodonPlus (DE3)-RIL competent cells (Agilent Technologies) were transformed with wild type or mutant construct of *ofd2*, grown in 2 l of LB medium containing 91.1 g/l D-sorbitol, 384 mg/l betaine hydrochloride, and 50 µg/ml kanamycin, at 37°C to A_600_∼1, chilled down to 16°C and induced with 1 mM isopropyl-β-D-thiogalactopyranoside at 16°C over night. Cells were sonicated five times for 20 s. (Vibra Cell sonicator, Model VC601, Sonics and Materials Inc.) in buffer A (50 mM Na_2_HPO_4_ (pH 8.0), 300 mM NaCl, 5 mM imidazole, 10 mM β-ME), cell debris removed and protein extract applied to a nickel-bound nitrilotriacetic acid (Ni-NTA) agarose column equilibrated with buffer A. After washing with buffer A containing 10 mM imidazole, proteins were eluted with buffer A containing 500 mM imidazole and fractions with Ofd2 were pooled and dialyzed (MWCO: 12-14000) against buffer B (10 mM Tris (pH 7.0), 50 mM NaCl, 10 mM β-ME) at 4°C for 3 h before applied to a Resource Q column. Proteins were eluted in buffer B containing a NaCl gradient of 50–1000 mM, fractions with Ofd2 were pooled and subjected to a second dialysis (MWCO: 12-14000), this time against buffer C (10 mM MES (pH 6.0), 50 mM NaCl, 10 mM β-ME) at 4°C for 3 h. The dialysate was applied to a Resource S column and proteins were eluted in buffer C containing a NaCl gradient of 50–1000 mM. Ofd2 purification was followed by SDS-PAGE.

### Succinate release assay

This method was adapted from Kaule and Günzler [40], but instead of using 2-oxo[1-^14^C]glutarate and measure the release of ^14^CO_2_, 2-oxo[5-^14^C]glutarate was used and the formation of [1-^14^C]succinate was measured. Purified wild type or mutant Ofd2 protein was incubated with or without calf thymus histones as substrates in a 50 µl reaction with a buffer containing 50 mM Tris (pH 7.5), 4 mM ascorbic acid, 80 µM FeSO_4_ and 160 µM 2-oxoglutarate (10% 2-oxo[5-^14^C]glutarate; MC-1393, Moravek Biochemicals) at 37°C for 30 min. Precipitation was performed by adding 25 µl of 0.16 M 2,4-dinitrophenylhydrazine in 30% HClO_4_ and 25 µl ice-cold carrier solution containing 20 mM succinate and 20 mM 2-oxoglutarate, followed by 30 min incubation at room temperature. Next, 50 µl 1 M 2-oxoglutarate was added, and the reactions were incubated for another 30 min at room temperature before full-speed centrifugation for 15 min. Finally, ∼80 µl of the supernatant were transferred to vials containing 2 ml Ultima Gold MV scintillation fluid (PerkinElmer), and the samples were counted for 10 min in a Tri-Carb 2900TR Liquid Scintillation Analyzer (Packard).

### Intracellular localization of Ofd2

Ofd2 was C-terminally tagged with a green fluorescent protein (GFP) starting with PCR amplification of *ofd2* ^+^ using the start primer 5′-CATGCCATGGTC**CATATG**TTGTATGAAAATATGAGC-3′ and stop primer 5′-CG**GGATCC**TCCAAAAACATGATTTTC-3′ without stop codon (restriction sites in bold letters). The gene was inserted into the *Nde*I and *Bam*HI restriction sites of a pREP41EGFPC vector [41] and transformed into *S. pombe* wild type cells, according to the protocol of the Sc EasyComp Transformation kit (Invitrogen). The transformation suspension was spread on selective minimal plates (EMM) [42] and the presence of GFP-tagged Ofd2 was later confirmed by PCR. Expression of the fusion protein Ofd2-GFP was controlled by the thiamine-repressible *nmt1* promoter and cultures of *S. pombe* wild type cells carrying the Ofd2-GFP plasmid were grown in EMM in the absence of thiamine and leucine at 30°C over night. The cells were supplied with fresh EMM and for nuclear staining the cells were incubated further at 30°C for 20 min in the presence of 0.02 µg/µl Hoechst 33342 (Invitrogen). Cells were examined using a Zeiss Axioplan 2 imaging microscope equipped with a Zeiss AxioCam HRc camera.

### Dot blot and far western analysis

Increasing amounts of the different calf thymus histones (0.125, 0.25, 0.5 and 1 µg) were manually dotted onto polyvinylidene fluoride (PVDF) membranes (Immobilon FL, Millipore). BSA (0.125, 0.25, 0.5 and 1 µg) was included as a negative control and Ofd2 (0.125, 0.25, 0.5 and 1 ng) as a positive control for the western detection. The membranes were blocked in 5% low fat dry milk (Bio-Rad) in phosphate buffered saline (PBS) prior to probing with Ofd2 (5 µg/ml in PBS/0.005% Tween 20) at 4°C over night with shaking. Another identical blot was treated similarly except that it was not probed with Ofd2. Primary antibody raised against recombinant Ofd2 (rabbit) was diluted in PBS/5% dry milk, whereas the secondary antibody, goat polyclonal anti-rabbit IgG (horseradish peroxidase (HRP); Abcam ab6721) was diluted in PBS/0.005% Tween 20. The membranes were treated with chemiluminescence reagent (Immun-Star WesternC, Bio-Rad) and protein complexes visualized in an Image Station (ChemiDoc XRS+, Bio-Rad). For far western analysis, unfractionated calf thymus histones (1.25 µg), recombinant human histones (rH2A, rH2B, rH3, rH4; 1 µg) and Ofd2 (5 ng) were separated on 12% SDS-PAGE in 1× MES buffer and transferred to PVDF membranes (iBlot, Invitrogen). The membranes were probed with Ofd2 (5 µg/ml in PBS/0.005% Tween 20) at room temperature for 1 hour and protein complexes detected as for dot blot analysis. A similar gel, except that 1 µg Ofd2 was loaded, was subjected to Coomassie Blue staining as a loading control.

### Bioinformatics analysis

The sequences of *S. pombe* Ofd2 and 63 homologous sequences from fungal and metazoan species were obtained from the NCBI protein sequence databases [43]. The sequences for two additional *Schizosaccharomyces* species, *S. cryophilus* and *S. octosporus*, were retrieved from the Broad Institute Schizosaccharomyces group database [44] (http://www.broadinstitute.org/annotation/genome/schizosaccharomyces_group). The sequences were aligned with Expresso [45] and manipulated in Jalview [46]. The main bulk of sequences were subsequently removed in order to give a reliable alignment of Ofd2, AlkB and human and fungal homologs. Structural disorder predictions were performed with the VSL1 algorithm [47] and DISOPRED2 [48]. The structural model of the Ofd2 core domain was derived from an *E. coli* AlkB template from Yu and Hunt [32] (Protein Databank identifier 3I3Q) and the illustration was generated with PyMOL [49].

## Supporting Information

Figure S1 **Western blot analysis of the Ofd2-GFP fusion protein.** Protein extracts were prepared from *S. pombe* cells overexpressing Ofd2-GFP or GFP alone by the glassbead method in a BeadBeater in 25 mM Hepes (pH 7.4), 0.1 mM EDTA, 100 mM KCl, 2 mM DTT and Protease Inhibitor Cocktail (CalBiochem Set IV). Extracts were run on 10% SDS-PAGE in 1× MOPS. The gels were transferred to PVDF membranes as described in Material and methods and probed with either Ofd2 antibody (left panel) or GFP antibody (right panel; Abcam Ab290). Two parallel extracts were made from Ofd2-GFP expressing cells and 20 µg was loaded, whereas for GFP, 4 µg was loaded. 5 ng recombinant Ofd2 was included as positive control for the Ofd2 antibody. Molecular weight marker (M) was MagicMark XP (Invitrogen).
(TIF)

Figure S2 **Ofd2 has no activity on methylated oligonucleotide substrates.** Increasing amounts of Ofd2 and *E. coli* AlkB were incubated with [^3^H]MNU-treated oligonucleotides in the presence of 2OG and Fe(II). When double-stranded substrates were used, the [^3^H]MNU-treated oligonucleotides were annealed to their respective (unmethylated) complementary strand. (**A**) Poly(dA) oligonucleotide (TAAAATAATAAATTAAA) and (**B**) AC-rich oligonucleotide (AAACAAAAACAAAAACAAA).
(TIF)

Figure S3 **The** ***ofd2*** **^−^ mutant is not sensitive to MMS exposure.** Cells were grown to mid-log phase, serially diluted and spotted onto YES plates without MMS (control) and with increasing doses of MMS. Four separate *ofd2* ^−^ mutants from the same transformation were compared to *S. pombe* wild type (FY526).
(TIF)

Methods S1 **Supporting materials and methods for experiments shown in supporting figures and “data not shown”.**
(DOC)
