# Supplementary figures and images for: Schizosaccharomyces pombe Ofd2 Is a Nuclear 2-Oxoglutarate and Iron Dependent Dioxygenase Interacting with Histones
Source: PLoS One. 2011 Sep 16;6(9):e25188. doi: 10.1371/journal.pone.0025188 (PMC3175000; doi:10.1371/journal.pone.0025188)

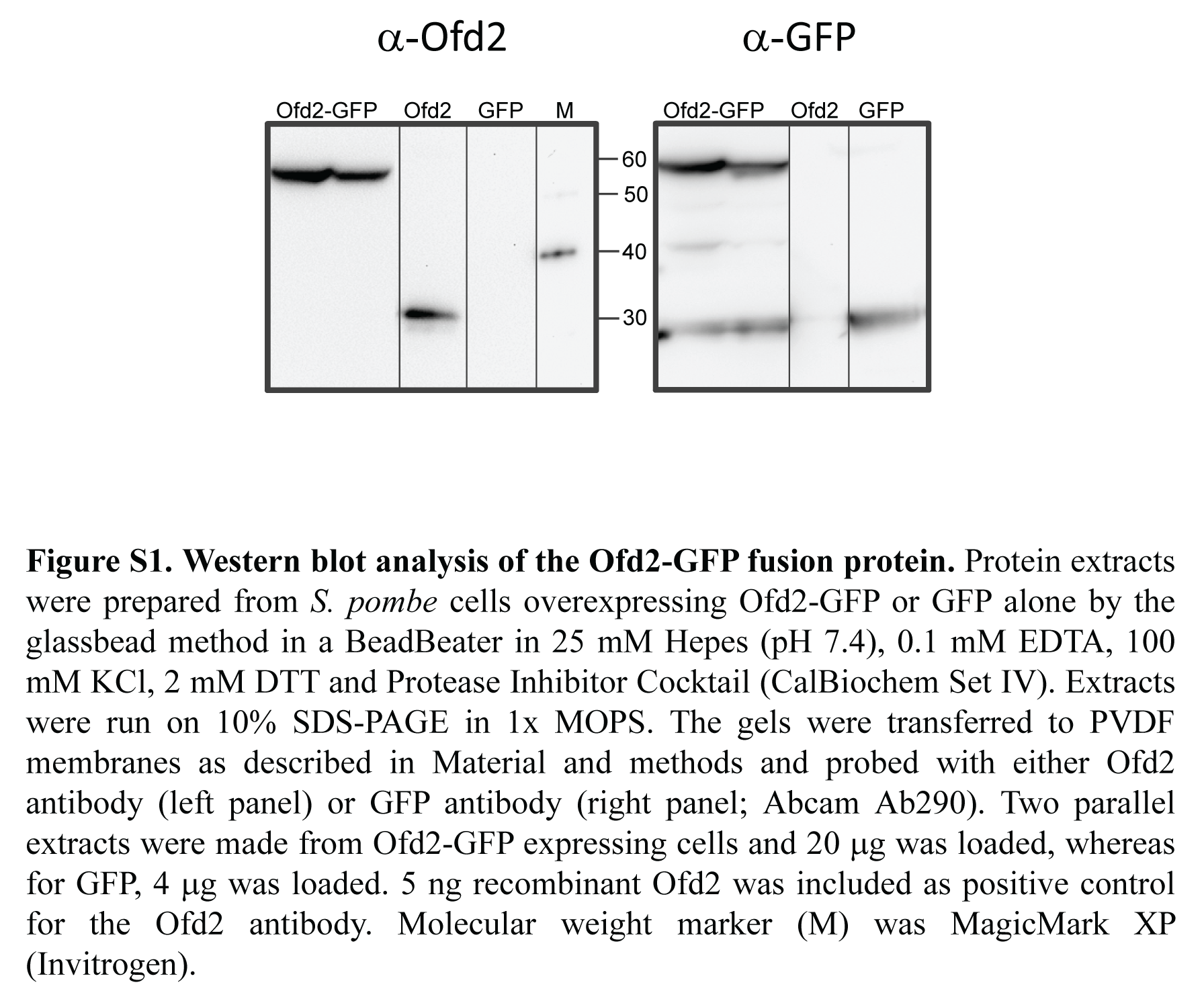

Supplement: Figure S1 — Western blot analysis of the Ofd2-GFP fusion protein. Protein extracts were prepared from S. pombe cells overexpressing Ofd2-GFP or GFP alone by the glassbead method in a BeadBeater in 25 mM Hepes (pH 7.4), 0.1 mM EDTA, 100 mM KCl, 2 mM DTT and Protease Inhibitor Cocktail (CalBiochem Set IV). Extracts were run on 10% SDS-PAGE in 1× MOPS. The gels were transferred to PVDF membranes as described in Material and methods and probed with either Ofd2 antibody (left panel) or GFP antibody (right panel; Abcam Ab290). Two parallel extracts were made from Ofd2-GFP expressing cells and 20 µg was loaded, whereas for GFP, 4 µg was loaded. 5 ng recombinant Ofd2 was included as positive control for the Ofd2 antibody. Molecular weight marker (M) was MagicMark XP (Invitrogen). (TIF) [file pone.0025188.s001.tif]

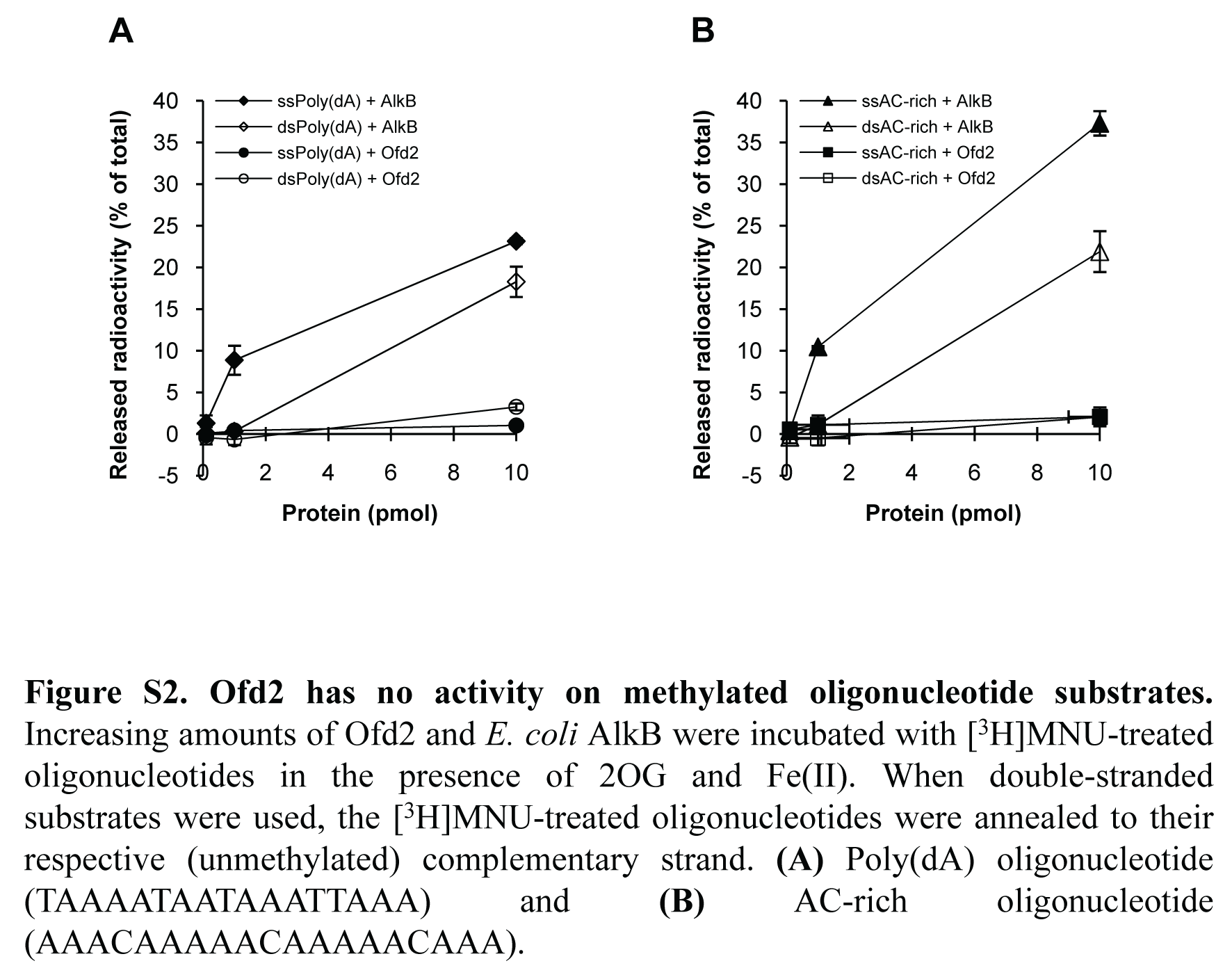

Supplement: Figure S2 — Ofd2 has no activity on methylated oligonucleotide substrates. Increasing amounts of Ofd2 and E. coli AlkB were incubated with [3H]MNU-treated oligonucleotides in the presence of 2OG and Fe(II). When double-stranded substrates were used, the [3H]MNU-treated oligonucleotides were annealed to their respective (unmethylated) complementary strand. (A) Poly(dA) oligonucleotide (TAAAATAATAAATTAAA) and (B) AC-rich oligonucleotide (AAACAAAAACAAAAACAAA). (TIF) [file pone.0025188.s002.tif]

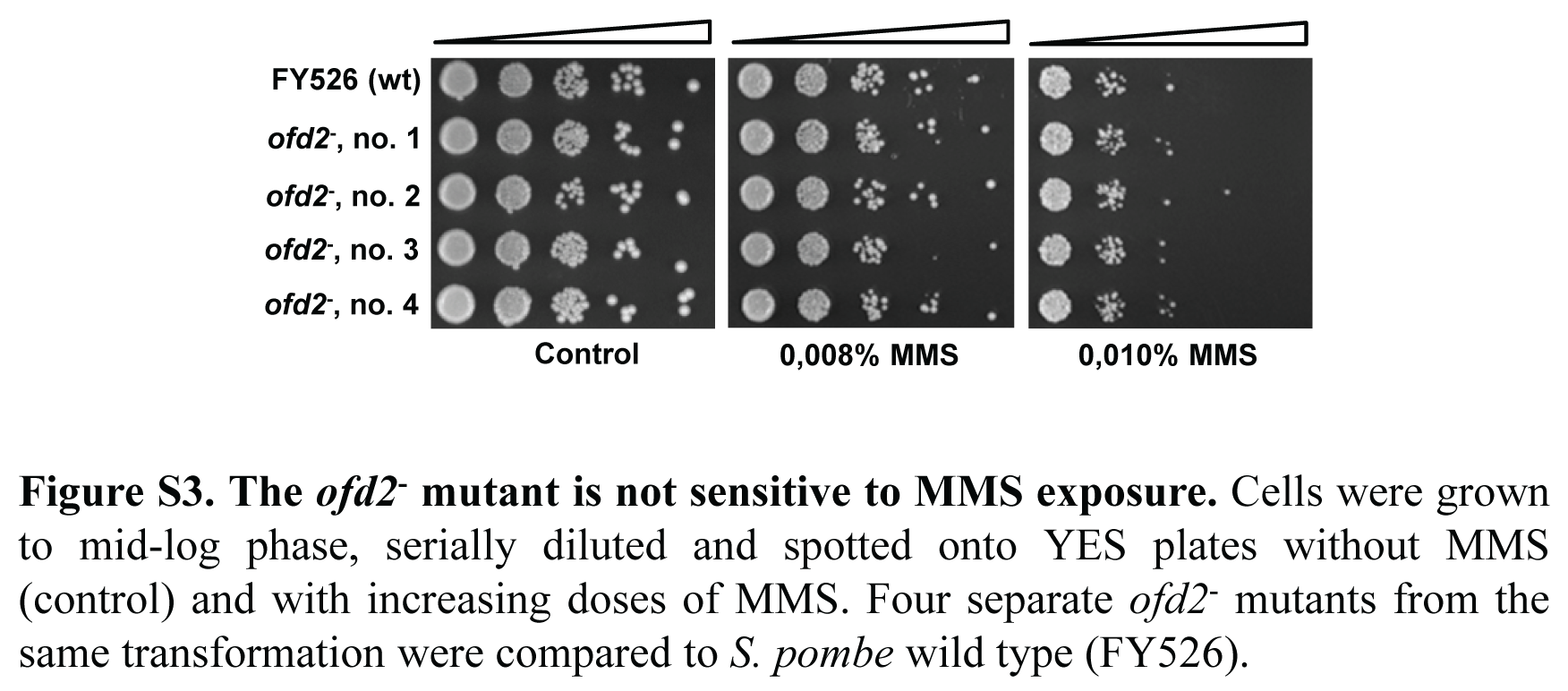

Supplement: Figure S3 — The ofd2 − mutant is not sensitive to MMS exposure. Cells were grown to mid-log phase, serially diluted and spotted onto YES plates without MMS (control) and with increasing doses of MMS. Four separate ofd2 − mutants from the same transformation were compared to S. pombe wild type (FY526). (TIF) [file pone.0025188.s003.tif]
