# Supplementary material for: Schizosaccharomyces pombe Ofd2 Is a Nuclear 2-Oxoglutarate and Iron Dependent Dioxygenase Interacting with Histones
Source: PLoS One. 2011 Sep 16;6(9):e25188. doi: 10.1371/journal.pone.0025188 (PMC3175000; doi:10.1371/journal.pone.0025188)
Supplement: Methods S1 — Supporting materials and methods for experiments shown in supporting figures and “data not shown”. (DOC) [file pone.0025188.s004.doc]

**Methods S1**

**Cultures and media**

The complete medium used was yeast extract medium supplemented with 225 mg/l adenine, uracil, histidine, leucine, arginine and lysine (YES). The minimal medium used in the 5-fluoroorotic acid (FOA) assay was pombe minimal medium with glutamate (PMG) [1] containing a uracil drop-out mix (225 mg/l of each amino acid - except 450 mg/l of leucine - 225 mg/l of adenine, 225 mg/l of myo-inositol, 45 mg/l para-amino benzoic acid). PMG(+all) contained 225 mg/l uracil in addition to what is described above, whereas PMG(+FOA) contained 125 mg/l uracil and 1 g/l 5-FOA (F5013, Sigma).

**Preparation of [^3^H]-methylated substrates**

DNA oligonucleotides (300 µg) was treated with *N*-[^3^H]methyl-*N*-nitrosourea as previously described [2]. For generation of double-stranded [^3^H]-methylated substrate, the single-stranded [^3^H]-methylated oligonucleotide was incubated with equal molar amount of non-methylated complementary strand for 2 min at 90°C and then slowly cooled to room temperature.

**Assay for oxidative demethylation of [^3^H]-methylated DNA**

[^3^H]-methylated DNA (approximately 0.1 µg containing 1000 d.p.m. label) was incubated in a 50 µl reaction mixture in the presence of varying amounts of Ofd2 as previously described [2].

***ofd2*^-^ disruption mutant**

*S. pombe* *ofd2*^+^ was cloned into the plasmid pQE-32 (QIAGEN). The *ofd2*^-^ disruption construct was generated by integrating the *LEU2* cassette of pINV1-GST [3] into the *Hin*dIII site of *ofd2*^+^. The *ofd2*::*LEU2* mutant strain was made by excising the *ofd2*::*LEU2* fragment from pQE-32 and transformation of *S. pombe* wild type cells, according to the protocol of the Sc EasyComp Transformation kit (Invitrogen).

**MMS survival assay**

*S. pombe* cells were grown in triplicates in YES medium until A_600_ was 0.6-0.8, serially diluted and spotted onto YES plates containing various concentrations of MMS. Plates were incubated at 30°C for 4 days.

**FOA spot assay**

Strains used in this assay contained the *ura4*^+^ reporter gene in four different heterochromatic loci: repeats of centromere 1 [*imr1*R(*Nco*I)::*ura4*^+^], central core of centromere 2 [*cen2*(*Sph*1)::*ura4*^+^], mating-type region [*mat3-M*(*Eco*RV)::*ura4*^+^] and ribosomal DNA [rDNA::*ura4*^+^] [4]. These strains, in combinations with either *ofd2* wt or *ofd2*^-^, were grown in YES medium until A_600_ was 0.6-0.8, serially diluted and spotted onto PMG(+all), PMG(-ura) and PMG(+FOA). Plates were incubated at 30°C for 3 days. Cells expressing the *ura4*^+^ gene cannot grow on plates containing FOA because FOA is converted to the toxic compound 5-fluorouracil when *ura4*^+^ is expressed.

**Supporting references**

1. Moreno S, Klar A, Nurse P (1991) Molecular genetic analysis of fission yeast Schizosaccharomyces pombe. Methods Enzymol 194: 795-823.

2. Falnes PO, Bjoras M, Aas PA, Sundheim O, Seeberg E (2004) Substrate specificities of bacterial and human AlkB proteins. Nucleic Acids Research 32(11): 3456-61.

3. Iacovoni JS, Russell P, Gaits F (1999) A new inducible protein expression system in fission yeast based on the glucose-repressed inv1 promoter. Gene 232: 53-58.

4. Bjerling P, Ekwall K, Egel R, Thon G (2004) A novel type of silencing factor, Clr2, is necessary for transcriptional silencing at various chromosomal locations in the fission yeast Schizosaccharomyces pombe. Nucleic Acids Res 32: 4421-4428.
